# AKT1 Loss Correlates with Episomal HPV16 in Vulval Intraepithelial Neoplasia

**DOI:** 10.1371/journal.pone.0038608

**Published:** 2012-06-07

**Authors:** Arucha L. Ekeowa-Anderson, Karin J. Purdie, Karen Gibbon, Carolyn R. Byrne, Jeffrey M. Arbeit, Catherine A. Harwood, Ryan F. L. O'Shaughnessy

**Affiliations:** 1 Centre for Cutaneous Research, Blizard Institute, Barts and the London School of Medicine and Dentistry, Queen Mary University of London, London, United Kingdom; 2 Department of Dermatology, Whipps Cross University Hospital, London, United Kingdom; 3 Division of Urologic Surgery, Washington University School of Medicine, St Louis, Missouri, United States of America; 4 Departments of Immunobiology and Dermatology, Institute of Child Health, University College London, London, United Kingdom; Karolinska Institutet, Sweden

## Abstract

Anogenital malignancy has a significant association with high-risk mucosal alpha-human papillomaviruses (alpha-PV), particularly HPV 16 and 18 whereas extragenital SCC has been linked to the presence of cutaneous beta and gamma–HPV types. Vulval skin may be colonised by both mucosal and cutaneous (beta-, mu-, nu- and gamma-) PV types, but there are few systematic studies investigating their presence and their relative contributions to vulval malignancy. Dysregulation of AKT, a serine/threonine kinase, plays a significant role in several cancers. Mucosal HPV types can increase AKT phosphorylation and activity whereas cutaneous HPV types down-regulate AKT1 expression, probably to weaken the cornified envelope to promote viral release. We assessed the presence of mucosal and cutaneous HPV in vulval malignancy and its relationship to AKT1 expression in order to establish the corresponding HPV and AKT1 profile of normal vulval skin, vulval intraepithelial neoplasia (VIN) and vulval squamous cell carcinoma (vSCC). We show that HPV16 is the principle HPV type present in VIN, there were few detectable beta types present and AKT1 loss was not associated with the presence of these cutaneous HPV. We show that HPV16 early gene expression reduced AKT1 expression in transgenic mouse epidermis. AKT1 loss in our VIN cohort correlated with presence of high copy number, episomal HPV16. Maintained AKT1 expression correlated with low copy number, an increased frequency of integration and increased HPV16E7 expression, a finding we replicated in another untyped cohort of vSCC. Since expression of E7 reflects tumour progression, these findings suggest that AKT1 loss associated with episomal HPV16 may have positive prognostic implications in vulval malignancy.

## Introduction

Human papillomaviruses (HPV) are a family of over 120 small double-stranded DNA viruses with mucosal or cutaneous epitheliotropism [1]. The mucosal alpha-papillomavirus (alpha-PV) genus contains high-risk HPV types, such as HPV 16 and 18, which are causal in cervical intraepithelial neoplasia (CIN) and cervical cancer [2]. Cutaneous beta-PVs are ubiquitous in skin and hair follicles. They are associated with cutaneous squamous cell carcinoma (SCC) development in the genodermatosis epidermodysplasia verruciformis and may also act as co-factors with ultraviolet radiation (UVR) in the pathogenesis of cutaneous SCC arising outside the context of EV, particularly in immunosuppressed individuals [3]. However recent RNA sequencing analysis of SCCs has put the role of beta-papillomaviridae into question [4]. Cutaneous mu-, nu- and gamma-PV genuses have also been implicated in causing benign and malignant humans skin lesions [3]. Although less common than cervical cancer, vulval squamous cell carcinoma (vSCC) and its precursor, vulval intraepithelial neoplasis (VIN), are increasing in incidence [5]. High-risk alpha-PVs may play an oncogenic role as approximately 60% of VIN are alpha-PV DNA positive [6], [7]. VIN may be classified into the usual or undifferentiated type (uVIN) associated with HPV and differentiated VIN (dVIN) associated with the chronic inflammatory dermatosis, lichen sclerosus (LS), and regarded as non-HPV mediated [8].

AKT1 is one of 3 isoforms of the AKT family of serine/threonine kinases. AKT1 induces protein synthesis and has a role in normal cellular growth. It also inhibits apoptosis and its dysregulation plays a significant role in several cancers [9]. Cervical cancer progression is accompanied by increased expression of the HPV E7 oncoprotein. E7 causes pleiotropic effects on cell cycle progression and inhibition of apoptosis [10]. We have previously shown that in extragenital cutaneous viral warts, HPV infection is associated with loss of expression of AKT1 in the upper granular layer of the epidermis [11]. This contrasts with the oncogenic E7 early gene from high-risk alpha-PVs such as HPV16 and beta-PVs which up-regulate a lower suprabasal AKT activity [12], [13]. Loss of AKT1 in non-genital warts causes changes in cornified envelope structure that we postulate are required for HPV virions to escape from the cornified envelope [11], [13]. The HPV types associated with this loss have not yet been clearly defined, although expression of the complete early region of HPV8, a cutaneous beta-PV, in mouse epidermis, or the expression of HPV8 E2 in keratinocyte organotypic cultures reduces AKT1 expression [11], [14].

The nature of the HPV infection in VIN remains less well-defined than in CIN, as does the mechanism by which HPV contributes to the risk of the lesion becoming an invasive SCC. We hypothesised that VIN have a higher prevalence of non-alpha-PV types compared to cervix and that combined effects of alpha and beta-PV types result in pathogenic mechanisms of VIN distinct from those in cervical dysplasia. In contrast, high-risk alpha papillomaviridae (HPV16), was the prevalent subtype in our VIN cohort. AKT1 loss correlated with episomal, high copy number HPV16 infection, while low copy number and integration events and high levels of the E7 oncoprotein correlated with normal levels of AKT1 expression.

## Materials and Methods

### Patients and samples

Clinical material was obtained from immunocompetent patients attending the dermatology and specialist vulval oncology clinics at Barts and the London NHS Trust. Routine histopathologic evaluation, hematoxylin-eosin, was performed on formalin-fixed lesional sections. Both formalin fixed sections and samples snap-frozen in liquid nitrogen and stored at −80°C were analysed in this study. Ethical approval for this investigation was obtained from the East London and City Health Authority local ethics committee and the study was conducted according to the Declaration of Helsinki Principles. All patients participating in the study provided written, informed consent. Cervical tissue sections were obtained from Biomax US (Rockville, US). Ear epidermal sections were obtained from 3 different 6 month old K14-HPV16 complete early region mice [15].

### Immunohistochemistry

Immunohistochemistry was performed on formalin fixed paraffin-embedded tissue sections using standard techniques. Primary antibodies used were as follows: rabbit anti-Akt1 (Cell Signaling Technologies, Danvers, USA) 1∶50; rabbit anti-keratin 1 (Covance, Cambridge, UK) 1∶500; rabbit anti-loricrin (Covance, Cambridge, UK) 1∶500; mouse anti HPV16E7 1∶50 (Santa Cruz Biotechnologies, Santa Cruz, US); Mouse anti HPV16E
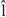
E4 (Santa Cruz Biotechnologies, Santa Cruz, US). Antibody detection was by the Elite Avidin-Biotin-Complex system and DAB (Vector Laboratories, Burlingame, US) using the appropriate conjugated secondary antibodies (Vector Laboratories, Burlingame, US). Sections were counterstained in haemotoxylin. Images were taken with a Leica DMLB microscope with a x20 (NA 0.4) objectives, using a Q imaging digital camera with QCapture software (Qimaging, Surrey, Canada).

### HPV detection and genotyping by degenerate and nested PCR and copy number evaluation

Mucosal alpha-PV genotyping was performed on both formalin fixed sections and samples snap-frozen in liquid nitrogen using a technique suitable for poor quality, degraded DNA. Other HPV genotyping and copy number analysis were restricted to samples snap-frozen in liquid nitrogen because of a prerequisite for good quality DNA. Detection of mucosal alpha-PV was performed by PCR using the SPF10 primer set [16], which amplifies a small fragment of 65 bp within the L1 open reading frame, with typing by reverse hybridisation assay (HPV SPF10 LiPA kit, Labo Bio-Medical Products, Rijswijk, Netherlands). Detection of mu/nu-PV and muco-cutaneous species 4 alpha-PV was performed using nested and degenerate PCR to the L1 open reading frame with typing by sequencing as described previously [17], specifically CN1F/CN1R primers nested within HVP2/B5 for mu/nu (including HPV1, 41 and 63) and CN2F/CN2R nested within HVP2/B5 for muco-cutaneous species 4 alpha-PV (including HPV 2,27,57). All beta-PV were detected using the PM primer set [18], which amplifies a 117 bp fragment from the E1 open reading frame, with typing by reverse hybridisation assay (RHA kit skin (beta) HPV detection system, Diassay, Rijswijk, Netherlands). HPV16 copy number was analysed according to the protocol of Weissenborn et al., [19].

### PCR analysis of HPV16 E1-E2 and E7 genomic regions

Standard PCR was performed on DNA from the frozen VINs to a region encompassing the E1 and E2 early gene region, Forward primer -5′-ATGGTACAATGGGCCTACGATAATG-3′, Reverse primer -5′-CGTCTGTGTTTCTTCGGTGC. Product size, 1522 bp, a second primer pair amplifying a 1.2 kb fragment of the Alpha-1 antitrypsin gene was used to confirm DNA integrity. Forward primer -5′ -CGACGAGAAAGGGACTGAAG, Reverse primer -5′ - GACAGCAACAGGCACAAAGA. E7 genomic PCR was performed on DNA extracted from paraffin embedded sections of the vSCCs to confirm the results of HPV SPF10 LiPA analysis. Forward primer 1 5′- ACTCTACGCTTCGGTTGTGC, reverse primer 1 - 5′ - TGCCCATTAACAGGTCTTCC. Product size 72 bp. Forward primer 2 5′ - ACAAGCAGAACCGGACAGAG, reverse primer 2 5′ - GCACAACCGAAGCGTAGAGT. Product size 76 bp.

### Statistical analysis

Correlations between viral presence, HPV copy number, integration status and AKT1 loss were determined using the Fisher's exact test. An unpaired T-test was used to determine significance of changes in HPV16 copy number. A Mann-Whitney U test was used to determine the association between between AKT1 expression and HPV16E7 immunohistochemistry score in the untyped samples.

## Results

### Expression of AKT1 is identical in vulval and non-genital skin

**Figure 1:**
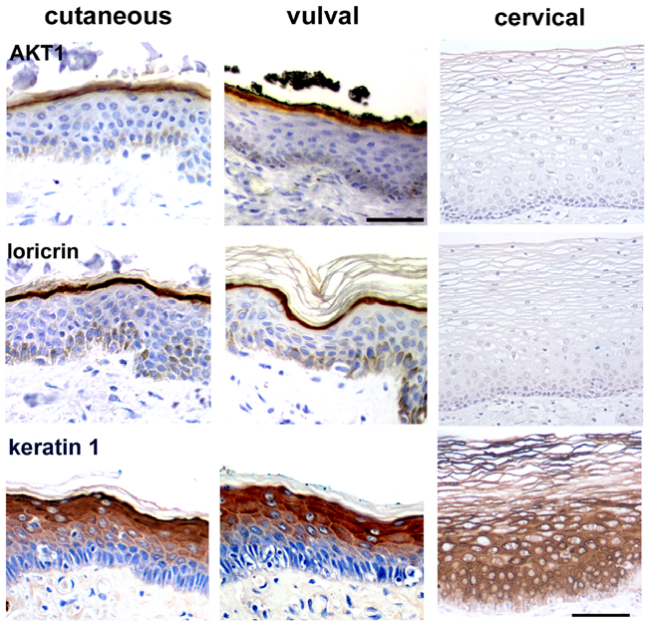
Keratinocyte differentiation marker expression in epidermis, vulva and cervix. Immunohistochemical analysis of AKT1, loricrin and keratin 1 expression in extragenital cutaneous epidermis, vulval external epithelium and cervix. Bar 50 µm.

We compared the expression of AKT1 and loricrin (a differentiation marker expressed in the upper epidermal granular layer) in normal extragenital cutaneous, cervical and vulval epithelia (Figure 1). Extragenital and vulval epidermis had identical expression patterns of AKT1 and loricrin, with expression of both proteins being restricted to the granular layer [13]. In contrast, neither AKT1 nor loricrin were detected in the upper cervical epithelium. Cytokeratin 1 was expressed in all suprabasal layers of all three epithelia. These findings suggest that the squamous keratinising epithelial component of vulval skin is similar to extragenital epidermis and may therefore be similarly susceptible to infection by cutaneous HPV types as well as alpha (mucosal) high risk HPV types.

### No correlation between either Beta-PV or HPV16 and AKT1 loss

**Figure 2:**
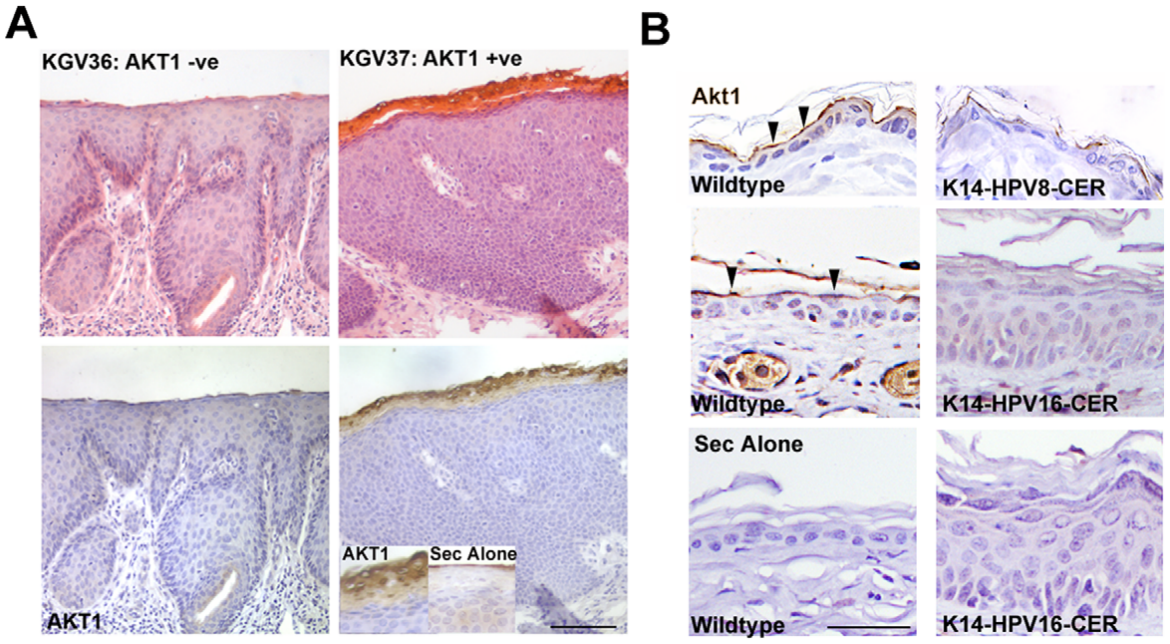
AKT1 expression is lost in a subset of VIN and the epidermis of the K14-HPV16 mouse. A. Histology and AKT1 expression in two representative VIN. Inset shows specific AKT1 expression associated with nucleated upper epidermal cells. Secondary alone control of upper epidermis is also shown. B. Immunohistochemical analysis of AKT1 in the dorsal epidermis of the K14-HPV8 complete early region (CER) and ear epidermis of the K14-HPV16 complete early region mouse and corresponding wildtype controls (wildtype). Arrowheads indicate AKT1 positivity in the granular layer of controls. Sec alone is a no primary antibody control for staining specificity. Bar (A–B) 50 µm.

We wanted to establish whether AKT1 is down-regulated similar to non-genital skin or up-regulated, potentially due to alpha-PV. We therefore analysed HPV types present in 14 snap-frozen high-grade VIN (Histological grade 2 and 3) (Table 1), correlating this with AKT1 loss. AKT1 expression was examined by immunohistochemistry ([13]; Figure 2A). AKT1 was lost in 6/14 (42%) of VIN, and HPV16 was present in 11/14 (78%) samples. Five of the 6 (83%) AKT1 negative VIN were HPV16 positive, while 6/8 (75%) of AKT1 positive VIN were HPV16 positive (n.s., Fishers exact test). Beta-PV types were detected in 2/14 (14%, one AKT1 positive, one AKT1 negative, Table 1). Although the RHA assay is non-quantitative, the weakness of the beta-PV signals compared to HPV16 signals was consistent with beta-PV being present at very low copy number (data not shown). Note that no other HPV types were detected by this analysis (data not shown). We concluded that beta-PV were unlikely to be significant aetiological agents in VIN.

**Table 1:** Summary of nested PCR analysis and sequencing for HPV16/18, and Beta-PV types.

	DIAGNOSIS	MY11_GP6	Mu/Nu	Alpha 4	Beta	Akt1	Copy No	E1-E2 PCR Positive	E1-E4	E7
**KGV02**	VIN3, AIN3	HPV16	−	+++	−	neg	7.7	yes	n.d.	n.d.
**KGV05**	VIN and LS	HPV16	−	+++	+	neg	10.9	yes	low	low
**KGV08**	VIN2	HPV16	−	+++	−	neg	5	yes	low	low
**KGV11**	VIN3	HPV16	−	+++	−	neg	0.36	no	high	high
**KGV29**	VIN3	-	−	−	−	neg	n.d.	n.d.	n.d.	n.d.
**KGV36**	LS and VIN	HPV16	−	−	−	neg	11.7	yes	n.d.	n.d.
**KGV01**	VIN3	HPV16	−	+++	−	pos	0.44	no	high	high
**KGV03**	VIN3	HPV16	−	+++	−	pos	0.51	no	high	high
**KGV10**	VIN	HPV16	−	+	+	pos	0.14	no	high	high
**KGV21**	VIN3	HPV16	−	−	−	pos	1.3	no	n.d.	n.d.
**KGV33**	VIN3	-	−	−	−	pos	n.d.	n.d.	n.d.	n.d.
**KGV37**	VIN	HPV16	−	+++	−	pos	0.2	no	n.d.	n.d.
**KGV40**	VIN and AIN	-	−	−	−	pos	n.d.	n.d.	n.d.	n.d.
**KGV48**	VIN	HPV16	−	−	−	pos	0.67	yes	high	high

-, sample is negative for this PV-type. AKT1 indicates the presence (pos) or absence (neg) of AKT1 expression in the epidermis of the VIN/vSCC. Copy no. indicates the HPV16 copy number per cell in each sample. Integrated? denotes the integration status of HPV16 based on the HPV16 genomic PCR. N.B. Several samples were PCR-positive for cutaneous alpha-PV species 4, however sequencing showed this was due to non-specific amplification of HPV16 in all cases; gamma and mu/nu types were not detected. E1-E4 and E7, summary of the immunohistochemistry for HPV16 E
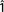
E4 and E7. n.d – not determined.

### HPV16 early gene expression reduces granular layer AKT1 expression

We used a transgenic mouse model to investigate whether the HPV16 early gene region causes AKT1 loss in the granular layer in a similar manner to the HPV8 complete early gene region [11]. Granular layer AKT1 expression was down-regulated in the non-lesional ear epidermis of the 6 month old mouse expressing the full length HPV16 complete early region under the control of the keratin 14 promoter ([15]; Figure 2B). The epidermis was hyperproliferative, unlike keratin 14-HPV8 early region transgenic mouse non-lesional epidermis [14]. Hyperproliferation in the K14-HPV16 mouse has been reported previously [15].

### AKT1 loss correlated with episomal HPV16 in VIN and predicted integration events correlated with increase in HPV16E7 expression

**Figure 3:**
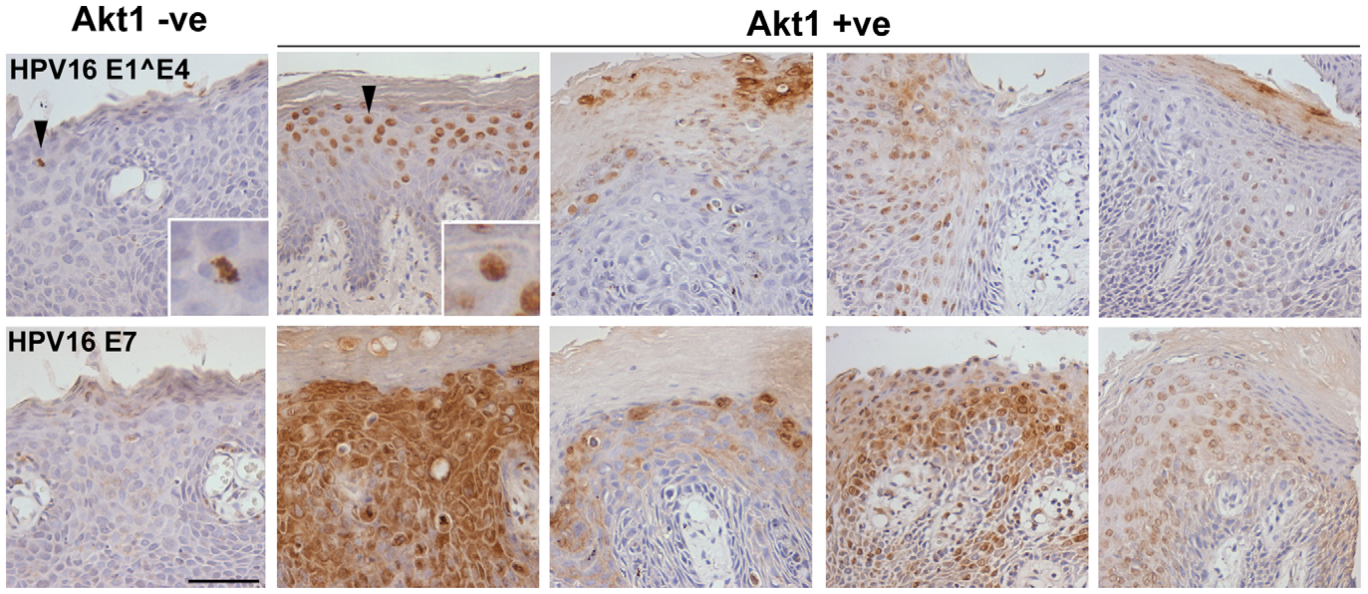
Early gene expression in VIN. Expression of the early genes 16E1∧E4 and 16E7 in a representative AKT1 negative (AKT1 −ve) and AKT1 positive (AKT1 +ve) VIN. Note the increased 16E7 expression in AKT1 positive VIN, and the change of 16E1∧E4 expression from perinuclear low expression in AKT1 negative VIN to high nuclear expression in AKT1 positive VIN (see insets). Bar 50µm.

To determine whether integration events correlated with changes in early gene expression, we examined 8 VIN for HPV16 E7 and E1∧E4 expression. HPV16 E7 is normally up-regulated after integration of HPV16 [20]. HPV16 E1∧E4 accumulates in mitochondria, clumping them in a region close to the nucleus [21] and on HPV16 integration is frequently truncated causing nuclear accumulation [22]. In HPV16 and AKT1 positive VIN, E7 expression was high and widespread not only in the nucleus, as has been reported previously in cervical cancer, but also in the cytoplasm in some VIN [23]. E1∧E4 expression was widespread and nuclear. In HPV16 positive and AKT1 negative VIN, E7 expression was lower and E1∧E4 was restricted to the perinuclear region in a few cells (Figure 3) suggesting again that AKT1 positivity correlates with life cycle events consistent with HPV16 integration in VIN.

**Figure 4:**
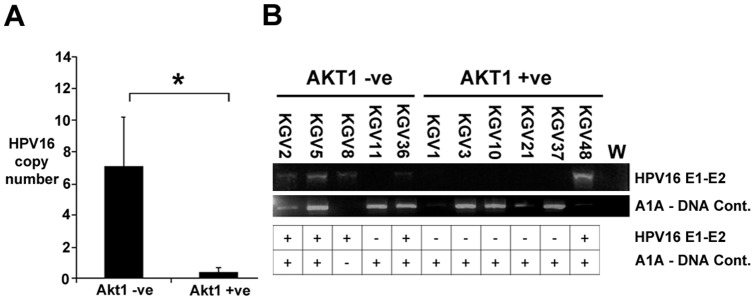
Copy number and integration analysis of VIN. A. Copy number analysis of HPV16 DNA in AKT1 −ve (p = 5) and AKT +ve (p = 6) VIN. *, p = 0.006 (unpaired T-test). B. PCR of DNA for a region encompassing the HPV16 E1 and E2 genes in the 14 VIN, and the relationship with AKT1 loss in the VIN. L. 250 bp DNA ladder, W, no DNA control.

As AKT1 loss was only detected in a subset of HPV16 positive VIN, we investigated whether this correlated with the physical status (episomal versus integrated) of the viral genome; we hypothesise that only episomal HPV16 is capable of lifecycle completion which concomitantly requires AKT1 down-regulation [11]. HPV16 copy number was increased on average 16-fold in AKT1 negative VIN (Figure 4A). The average copy number per cell in AKT1 positive VIN was less than 1, suggestive of genome integration [24]. We tested for HPV16 integration in the 11 HPV16 positive VIN by PCR using primers encompassing the E1 and E2 genes of HPV16 (Figure 4B), whose loss correlates with an integrated viral genome [25]. PCR product was detected in 4/5 (80%) of the AKT1 negative, HPV16 positive VIN tested, compared with 1/6 (17%) of the AKT1 positive, HPV16 positive VIN (p = 0.08, Fishers Exact Test). An alpha-1 antitrypsin PCR product was detected in all E1-E2 negative samples, suggesting that these negative results were not due to lack of DNA integrity. Although not reaching significance, this suggested that HPV16 was more likely to be integrated in the AKT1 positive VIN.

### AKT1 loss correlated with low expression of HPV16 E7 in a vSCC cohort

**Figure 5:**
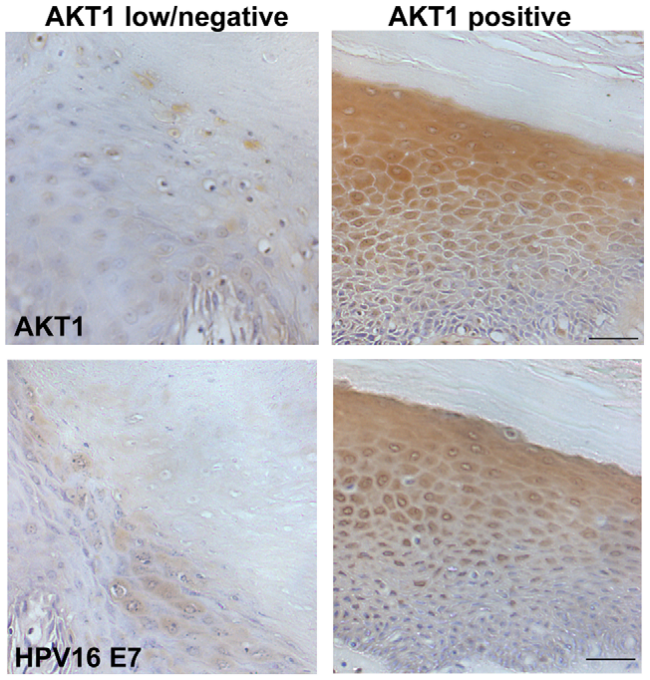
AKT1 expression and HPV16E7 expression in vSCC cohort. Representative data of immunohistochemistry of the archive vSCC cohort. AKT1 expression and HPV16E7 expression were analysed by immunohistochemistry. Loss of AKT1 associated with low HPV16E7 levels, while maintained AKT1 correlated with high HPV16E7 expression. Bar 50 µm.

To determine the diagnostic significance that AKT1 loss may have in assessing the progression or severity of VIN, we examined another series of 30 paraffin embedded vSCC for both AKT1 loss and HPV16 E7 protein expression. AKT1 loss occurred in 12/30 (40%) of the samples consistent with the first set of samples. Again, as with the first set of samples, AKT1 negative vSCC had significantly lower E7 staining scores than AKT1 positive VIN (p = 0.003, Mann-Whitney U test, Figure 5, and Table S1).

## Discussion

This is the first study to examine the prevalence of both cutaneous and mucosal HPV in vulval malignancy and to correlate the status of HPV16 with AKT1 expression. In a cohort of VIN, we found that that AKT1 loss correlated with the presence of high copy number, episomal HPV16. When AKT1 and HPV16 were both present, this was associated with integration events, with a high level of HPV16E7 expression and altered localisation of the EÎE4 protein. Our data suggests a model in which HPV16 expression in the external vulval epithelium is episomal at first. AKT1 is down-regulated at this time by the E2 protein [11], to weaken the cornified layer to allow for continued rounds of papillomavirus infection. Subsequently the HPV16 genome is disrupted by integration, normally at the site of the E1 and E2 early genes, restoring AKT1 expression. These data and analysis of our untyped vSCC cohort indicated that restoration of AKT1 expression correlated with up-regulation of HPV16 E7.

### HPV16 are highly prevalent HPV in vulval malignancy, while beta papillomaviruses do not play a role in malignant progression

The prevalence of different HPV types by analysis of HPV DNA in vulval SCC differs significantly from those reported in previous studies of SCC at extragenital cutaneous sites by our group and other researchers using the same and alternative methodologies [3], [26]. Compared with our previous findings using the same methodology in cutaneous SCC and carcinoma in situ which is equivalent to VIN [27], there was a higher prevalence of mucosal alpha-PVs (79% in vulva compared with 8.5% cutaneous CIS/SCC% in extragenital skin). Beta-PVs prevalence was much lower in the vulva (14% versus 57.4% respectively). Our study suggests that based on the relatively low amounts of beta-HPV DNA detected by the reverse hybridisation assays, beta-HPV types do not play a significant role in vulval malignancy, consistent with previous analyses of beta-HPV presence in anogential plucked hairs [28].

### Episomal HPV16 correlates with AKT1 loss – Significance for HPV16 lifecycle in external epithelia

Although the expression of the HPV16 complete early region in epidermis can cause carcinogenesis [15], whether or not HPV16 can progress through lifecycle and produce virions in external (cutaneous) epithelia has yet to be determined. The data presented here suggests that HPV16 is able to progress through stages of its lifecycle, and can be retained episomally in the epidermis, as demonstrated indirectly by the loss of AKT1 and directly by the detection of the intact HPV16 E1-E2 genomic region. AKT1 expression was lost in 88% of non-genital warts [11] while in this study AKT1 was lost in 42% of VIN. This suggests that HPV16 more readily integrates in external epithelia, than other cutaneous resident viruses. We would therefore expect HPV16 infected external epithelia to more rapidly become malignant. Consistent with this concept, the mean time of tumour formation in the K14-HPV16 complete early region mouse is 3 fold less than that of the K14-HPV8 complete early region mouse [14], [15].

Average episomal HPV16 copy number was less than 10 per cell in the whole of the VIN cohort, compared to CIN where the mean copy number is over 2000 [29]. In high grade CIN, there is high HPV16 copy number as well as an increased frequency of integrants (40%,[30]). In contrast, in high grade VIN very low HPV16 copy number per cell correlated with our hallmarks of integration – nuclear E1-E4 expression, high E7 expression and Akt1 retention. The integration frequency in high grade VIN was higher than in high grade CIN–54% in our typed cohort and, based on AKT1 expression, 60% in our untyped cohort. One possible interpretation of these data is that HPV16 integrates more frequently as it is not able to efficiently produce high copy number virus in external keratinocytes.

In the cervix during progression to CIN and SCC, the expression of E7 is widespread and nuclear [20]. However in the external vulval epithelium, we observed cytoplasmic staining of HPV16 E7. Although there is little or no data on normal expression of cutaneous HPV E7, it may be that the expression pattern seen for HPV16E7 in VIN is normal. Transport of the E7 protein from the nucleus to the cytoplasm is mediated by a novel Ran-dependent nuclear export [23]. It is possible that in the epidermis, this export pathway is more prevalent than nuclear import, leading to increased cytoplasmic E7 expression in the epidermis. It would be interesting to examine the differences in gene expression between keratinocytes expressing nuclear HPV16E7 and cytoplasmic HPV16E7 to understand how this may relate to differences in HPV16 lifecycle and integration in the external vulval epithelium versus the cervix.

In summary, we show that cutaneous beta-PV are not significantly associated with vulval neoplasia, and that HPV16, when episomal, can reduce AKT1 expression. We propose that AKT1 expression status may therefore represent a potential prognostic biomarker in VIN. However, a larger study monitoring AKT1 expression with integration status and viral load over time would be required to determine whether AKT1 loss does indeed correlate with prognosis.

## Supporting Information

Table S1 **Summary of AKT1 and E7 antibody staining and L1 and E7 PCR for HPV16.** Summary of Immunohistochemistry and PCR on the vSCC cohort. AKT1 staining and PCR are scored positive or negative as in table 1. HPV16 E7 immunohistochemistry was scored from 0–3, where 0, is no staining, while 3 is high and widespread staining throughout the sample. PCR analysis of both HPV16L1 and two regions of HPV16E7 showed conflicting data and therefore HPV status could not be reliably ascertained using this method.
(DOC)
