# Supplementary material for: AKT1 Loss Correlates with Episomal HPV16 in Vulval Intraepithelial Neoplasia
Source: PLoS One. 2012 Jun 7;7(6):e38608. doi: 10.1371/journal.pone.0038608 (PMC3369856; doi:10.1371/journal.pone.0038608)
Supplement: Table S1 — Summary of AKT1 and E7 antibody staining and L1 and E7 PCR for HPV16. Summary of Immunohistochemistry and PCR on the vSCC cohort. AKT1 staining and PCR are scored positive or negative as in table 1. HPV16 E7 immunohistochemistry was scored from 0–3, where 0, is no staining, while 3 is high and widespread staining throughout the sample. PCR analysis of both HPV16L1 and two regions of HPV16E7 showed conflicting data and therefore HPV status could not be reliably ascertained using this method. (DOC) [file pone.0038608.s001.doc]

Table S1

| **Sample** | **Akt1** | **HPV16 E7 ihc** | **HPV16 L1** | **HPV16 E7 1** | **HPV16 E7 2** |
| --- | --- | --- | --- | --- | --- |
| **vSCC11** | neg | 0 | neg | neg | neg |
| **vSCC13** | neg | 2 | neg | neg | neg |
| **vSCC15** | neg | 1 | HPV16 | neg | neg |
| **vSCC16** | neg | 1 | neg | neg | neg |
| **vSCC17** | neg | 0 | HPV16 | neg | neg |
| **vSCC19** | neg | 0 | neg | Positive | neg |
| **vSCC20** | neg | 1 | HPV16 | neg | neg |
| **vSCC22** | neg | 0 | HPV16 | neg | neg |
| **vSCC24** | neg | 0 | neg | neg | neg |
| **vSCC27** | neg | 1 | HPV16 | neg | neg |
| **vSCC6** | neg | 1 | HPV16 | Positive | neg |
| **vSCC7** | neg | 0 | HPV16 | neg | Positive |
| **vSCC1** | positive | 2 | neg | neg | neg |
| **vSCC10** | positive | 2 | neg | neg | Positive |
| **vSCC12** | positive | 0 | neg | Positive | neg |
| **vSCC14** | positive | 2 | neg | neg | neg |
| **vSCC18** | positive | 1 | HPV16 | neg | neg |
| **vSCC2** | positive | 2 | neg | neg | neg |
| **vSCC21** | positive | 2 | neg | neg | neg |
| **vSCC23** | positive | 1 | neg | neg | neg |
| **vSCC25** | positive | 0 | HPV16 | neg | neg |
| **vSCC26** | positive | 2 | neg | neg | neg |
| **vSCC28** | positive | 3 | HPV16 | neg | neg |
| **vSCC29** | positive | 2 | neg | Positive | neg |
| **vSCC3** | positive | 3 | neg | neg | neg |
| **vSCC30** | positive | 0 | neg | neg | neg |
| **vSCC4** | positive | 2 | HPV16 | neg | positive |
| **vSCC5** | positive | 3 | HPV16 | neg | neg |
| **vSCC8** | positive | 2 | neg | neg | neg |
| **vSCC9** | positive | 2 | HPV16 | neg | Positive |
